# Effect of pregnancy planning on maternal and neonatal outcomes in women with Type 1 diabetes

**DOI:** 10.1111/dme.13398

**Published:** 2017-07-12

**Authors:** A. C. Wotherspoon, I. S. Young, C. C. Patterson, D. R. McCance, V. A. Holmes, the Diabetes and Pre‐eclampsia Intervention Trial (DAPIT) Study Group

**Affiliations:** ^1^ Centre for Public Health School of Medicine, Dentistry and Biomedical Sciences, Queen's University Belfast Belfast UK; ^2^ Regional Centre for Endocrinology and Diabetes Royal Victoria Hospital Belfast UK

## Abstract

**Aims:**

To assess the effect of pregnancy planning on maternal and neonatal outcomes in women with Type 1 diabetes.

**Methods:**

Pregnancy planning was assessed retrospectively in a cohort of women who participated in the Diabetes and Pre‐eclampsia Intervention Trial (DAPIT). Pregnancy planning was determined based on self‐report as to whether pregnancy was planned or unplanned. The effect of pregnancy planning on maternal and neonatal outcomes was examined, controlling for confounding variables.

**Results:**

A total of 747 women were included in the study, of whom 39% considered their pregnancy unplanned. Characteristics associated with unplanned pregnancy included being younger (*P*<0.001), being a current smoker (*P*<0.001), being from a lower social class (*P*<0.001) and having higher HbA_1c_ values prior to and throughout pregnancy (*P*≤0.005). Significantly fewer women with unplanned vs planned pregnancies received pre‐pregnancy counselling (24% vs 64%; *P*<0.001). Infants of women with unplanned pregnancies were more likely to be small for gestational age (<5^th^ centile; *P*=0.004), to be admitted to the neonatal care unit (*P*=0.001) and to have a longer stay in hospital (*P*=0.01). Outcomes did not differ between the groups in relation to pre‐eclampsia, congenital malformations or a composite adverse outcome.

**Conclusions:**

Risks associated with diabetes in pregnancy need to be highlighted to all women, their partners and families, and healthcare professionals. Further research is required to determine if these groups are fully aware of the risks associated with diabetes in pregnancy.

## What's new?

- Being young, a smoker and from a lower social class were associated with unplanned pregnancy.
- Outcomes that were associated with unplanned pregnancy were very low birth weight and greater neonatal and maternal care requirements post‐delivery
- Poor preconception counselling rates among women who did not plan pregnancy suggests an urgent need for strategies to ensure all women receive preconception counselling, regardless of pregnancy intention.

**What's new?**

- Being young, a smoker and from a lower social class were associated with unplanned pregnancy.
- Outcomes that were associated with unplanned pregnancy were very low birth weight and greater neonatal and maternal care requirements post‐delivery
- Poor preconception counselling rates among women who did not plan pregnancy suggests an urgent need for strategies to ensure all women receive preconception counselling, regardless of pregnancy intention.

## Introduction

Approximately half of women in the UK describe their pregnancy as unplanned or are ambivalent towards it 1. There are a range of behaviours that are known to be detrimental during pregnancy, including smoking and alcohol use 2, 3, many of which are associated with pregnancies that are unplanned. Such behaviours may contribute to the increased risks associated with unplanned pregnancy 2, 4. Planning a pregnancy is considered particularly important for women with diabetes, as they are at increased risk of a number of adverse pregnancy outcomes, including fetal malformation, stillbirth and pre‐eclampsia 5, 6. Poor glucose control in the preconception period and early pregnancy has been linked to an increased risk of adverse outcomes 7.

From adolescence, it is recommended that all women with diabetes, regardless of pregnancy intention, receive preconception counselling 8. This is a discussion between women and their healthcare professionals about the importance of planning for pregnancy and should inform women of the need for pre‐pregnancy care prior to conception. Pre‐pregnancy care is specialist care delivered by the multidisciplinary diabetes care team to help ensure a woman is prepared for pregnancy, and includes optimization of glycaemic control 8, the prescription of high‐dose folic acid supplements (5 mg) and review of their current medications.

Despite these recommendations, ~60% of women with diabetes still do not plan their pregnancy 9, 10, and enter pregnancy unprepared, increasing their risk of adverse outcomes. The Confidential Enquiry into Maternal and Child Health (CEMACH) reported an increased risk of poor pregnancy outcomes in women with unplanned pregnancies 9. The effect of pre‐pregnancy care on adverse outcomes in women with diabetes has been studied in some detail. Evidence shows that attending pre‐pregnancy care is associated with decreased HbA_1c_ levels in early pregnancy and with a reduced incidence of adverse outcomes, including congenital malformations, preterm birth and perinatal mortality 11, 12.

The aim of the present study was to assess the effect of pregnancy planning on maternal and neonatal outcomes in women with Type 1 diabetes.

## Participants and methods

A total of 747 women from the Diabetes and Pre‐eclampsia Intervention Trial (DAPIT) were included. DAPIT was a multi‐centre randomized double‐blind placebo‐controlled trial to investigate the use of antioxidants (vitamins C and E) for the prevention of pre‐eclampsia in women with Type 1 diabetes 13. Pregnancy planning was determined as part of a patient questionnaire completed by each woman at study randomization. Women were asked if their pregnancy was planned, with the response categorized as ‘yes’, ‘no’ or ‘not known’. Women were also asked if they had received pre‐pregnancy counselling (yes or no option), described to women as structured advice about the need to maintain good blood glucose control and healthy lifestyle (with respect to diet, exercise, BMI, smoking status and alcohol consumption) before trying to become pregnant, including the need to take folate supplements. This information was not independently confirmed or cross‐checked against clinical records.

Other maternal characteristics collected at baseline included BMI, smoking status, alcohol consumption and social class. Social class was classified according to occupation of the main earner in the household, based on the 1990 classifications 14. Each class was defined by occupation as: I, professional; II, managerial and technical; IIIN, skilled non‐manual; IIIM, skilled manual; IV, partly skilled; and V, unskilled.

Maternal and neonatal outcomes were collected as part of DAPIT and included pre‐eclampsia, gestational hypertension, eclampsia, fetal death, fetal malformation, caesarean section delivery, birth weight, admission to neonatal care unit, maternal stay in hospital and failure to attend a 6‐week postnatal visit. Pre‐eclampsia was defined as gestational hypertension with proteinuria, in accordance with international guidelines 15, 16. Gestational hypertension was defined as two diastolic blood pressure readings of ≥90 mmHg at least 4 h apart, or one reading of at least 110 mmHg after 20 weeks’ gestation. Proteinuria was defined as a result of at least 1+ for dipstick analysis of a midstream specimen on at least two occasions or 300 mg urinary protein per 24‐h period 13. Late fetal loss was defined as a baby born dead at a gestational age of 20–23 weeks. Neonatal death was categorized into early (death within the first 6 complete days of life) and late (death at age 7–27 completed days of life). Stillbirth was categorized into antepartum (fetal death occurring before labour at ≥24 weeks’ gestation) and intrapartum (fetal death occurring during labour at ≥24 weeks’ gestation). Birth weight centile was calculated from customized birth weight charts 17. Small for gestational age (SGA) was defined as birth weight below the 5^th^ centile and below the 10^th^ centile, while large for gestational age (LGA) was defined as birth weight above the 90^th^ centile. Informed written consent was obtained and the West Midlands Multicentre Ethics Research Committee provided ethical approval for DAPIT (MREC 02/7/016). DAPIT was carried out in accordance with the Declaration of Helsinki.

### Statistical analysis

Group comparisons were performed using chi‐squared and independent‐samples *t*‐tests. Logistic regression analysis was used to identify outcomes that were associated with unplanned pregnancies. A number of infrequent outcomes were combined *a priori* to create a composite adverse outcome: miscarriage; late fetal loss; stillbirth; neonatal death; termination; and major fetal malformation. All outcomes were adjusted for smoking, social class, maternal age, BMI, parity and centre. All statistical analyses were performed using spss version 20 (IBM Corp, Armonk, NY, USA).

## Results

### Characteristics

The maternal characteristics of women with planned and unplanned pregnancy are summarized in Table 1. Of the 747 women included in the study, 455 (60.9%) described their pregnancy as planned and 292 (39.1%) as unplanned. Women who reported having an unplanned pregnancy were significantly younger and more likely to smoke compared with women who had a planned pregnancy (*P*<0.001). Women with unplanned pregnancies tended to be in a lower social class (*P*<0.001) and to book later at their first antenatal visit than those who planned their pregnancy (*P*=0.01). Women with planned pregnancies were significantly more likely to have received pre‐pregnancy counselling than women with unplanned pregnancies (*P*<0.001). Those with unplanned pregnancies had significantly higher HbA_1c_ values 6 months prior to and throughout pregnancy, compared with those with planned pregnancies (*P*≤0.005). A significantly higher proportion of women who planned their pregnancy were taking folic acid prior to conception than those who did not plan pregnancy (*P*<0.001).

**Table 1:** Maternal characteristics of women with planned and unplanned pregnancies

Characteristic	Planned pregnancy	Unplanned pregnancy	*P*
	*n*_max_=455	*n*_max_=292	
Age, years	30.9 (4.8)	27.6 (6.3)	<0.001
Gestational age at first antenatal visit, weeks	8.6 (2.7)	9.1 (2.9)	0.01
BMI, kg/m^2^	27.5 (4.6)	27.4 (4.7)	0.80
Woman's ethnic origin non‐white, *n* (%)	13 (3)	10 (3)	0.66
Marital status, *n* (%)			
Married	366 (74)	104 (36)	
Cohabiting	96 (21)	90 (31)	<0.001
Never married	15 (3)	85 (29)	
Social class, *n* (%)			
Professional/managerial and technical occupations (I,II)	222 (49)	79 (27)	
Skilled, partly‐skilled and unskilled occupations (III,IV,V)	202 (46)	160 (55)	<0.001
Not known/not classified	31 (7)	53 (18)	
≤12 years in education, *n* (%)	155 (34)	135 (47)	<0.001
Duration of diabetes, years	14.8 (8.2)	14.1 (8.2)	0.25
Current smoker, *n* (%)	67 (15)	78 (27)	<0.001
Alcohol use, *n* (%)			
Never/stopped before pregnancy	255 (56)	154 (53)	
Stopped during pregnancy	150 (33)	150 (36)	0.66
Current	50 (11)	32 (11)	
Primiparous, *n* (%)	215 (47)	154 (53)	0.14
Abnormal renal status before this pregnancy*, *n* (%)	22 (5)	19 (7)	0.31
Retinal status before this pregnancy, *n* (%)			
Normal	322 (72)	210 (74)	
Background retinopathy	84 (19)	55 (19)	0.60
Abnormal**	39 (9)	19 (7)	
Recall receiving pre‐pregnancy counselling, *n* (%)	292 (64)	70 (24)	<0.001
HbA_1c_, mmol/mol			
Pre‐pregnancy (≤6 months prior)	61 (16)	74 (21)	<0.001
Randomisation (8–22 weeks’ gestation)	52 (12)	59 (12)	<0.001
34 weeks’ gestation	48 (7)	50 (8)	0.005
HbA_1c_, %			
Pre‐pregnancy (≤6 months prior)	7.7 (1.5)	8.9 (1.9)	
Randomisation (8–22 weeks’ gestation)	7.0 (0.8)	7.5 (1.1)	
34 weeks’ gestation	6.5 (0.6)	6.7 (0.8)	
Proportion with HbA_1c_ ≤50 mmol/mol, *n* (%)			
Pre‐pregnancy (≤6 months prior)	88 (25)	18 (9)	<0.001
Randomization (8–22 weeks’ gestation)	165 (41)	67 (27)	<0.001
34 weeks’ gestation	225 (69)	105 (58)	0.01
Systolic blood pressure at randomisation (8–22 weeks’ gestation), mmHg	119.2 (11.9)	118.4 (12.0)	0.35
Diastolic blood pressure at randomization (8–22 weeks’ gestation), mmHg	75.1 (8.4)	73.9 (8.7)	0.05
Taking folic acid prior to conception, *n* (%)	325 (72)	29 (10)	<0.001

Data are mean (sd) unless otherwise stated.

*Includes microalbuminuria, macroalbuminuria and 24h urinary protein >3 g/24 h.

**Includes proliferative retinopathy, maculopathy, previous vitrectomy and blindness.

### Maternal and neonatal outcomes

Maternal and neonatal outcomes are reported in Table 2. Gestational age at delivery was significantly lower in women with unplanned vs planned pregnancies (*P*=0.02).

**Table 2:** Maternal and fetal outcomes of women with planned and unplanned pregnancies

Outcome	Planned pregnancy (*n*=455)	Unplanned pregnancy (*n*=292)	Odds ratio (95%CI) for unplanned vs planned	
	*n*/*N* (%)	*n*/*N* (%)	Unadjusted	Adjusted†
Pre‐eclampsia	74/448 (17)	49/286 (17)	1.05 (0.70, 1.55)	1.23 (0.77, 1.95)
Gestational hypertension	53/448 (12)	30/286 (11)	0.87 (0.54, 1.40)	0.76 (0.45, 1.30)
Eclampsia	0/447 (0)	3/286 (1)	–	–
Caesarean delivery	307/454 (68)	200/286 (69)	1.04 (0.76, 1.43)	1.38 (0.96, 1.98)
Foetal death	12/449 (3)	6/284 (2)	0.79 (0.29, 2.12)	0.70 (0.24, 2.05)
SGA <5^th^ centile	13/446 (3)	24/284 (9)	3.08 (1.54, 6.14)**	3.10 (1.42, 6.75)**
SGA <10^th^ centile	26/446 (6)	31/284 (11)	1.98 (1.15, 3.41)*	1.68 (0.91, 3.11)
LGA >90^th^ centile	230/446 (52)	149/284 (53)	1.04 (0.77, 1.40)	1.19 (0.84, 1.68)
Major fetal malformation	15/454 (3)	13/291 (5)	1.37 (0.64, 2.92)	0.96 (0.41, 2.24)
Adverse outcome¶	30/455 (7)	26/292 (9)	1.39 (0.80, 2.39)	1.06 (0.57, 1.95)
Admission to neonatal intensive care unit	218/436 (50)	178/277 (64)	1.80 (1.32, 2.45)**	1.84 (1.28, 2.66)**
Infant stayed >10 days in hospital	65/434 (15)	64/277 (23)	1.71 (1.16, 2.51)**	1.75 (1.13, 2.70)*
Did not attend 6‐week postnatal visit	34/436 (12)	27/275 (15)	1.35 (0.88, 2.07)	1.15 (0.69, 1.93)

SGA, small for gestational age; LGA, large for gestational age.

†Odds ratio (95% CI) from logistic regression adjusted for smoking, social class, maternal age, BMI, parity and centre.

*P<0.05, **P<0.01.

¶Includes miscarriage, late fetal loss, stillbirth, neonatal deaths, termination and major malformation.

Rates of pre‐eclampsia and gestational hypertension did not differ significantly between the two groups. There were no significant differences between planned and unplanned pregnancies with regard to type of delivery, with similar proportions having vaginal and caesarean section deliveries.

There was a significant difference in birth weight between planned and unplanned pregnancies (*P*=0.008). This difference persisted after correction for gestational age; infants of women with unplanned pregnancies were more likely to be SGA, whether defined by the 5^th^ centile (*P*=0.001) or the 10^th^ centile *(P*=0.01). After adjusting for other covariates, SGA defined by the 10^th^ centile was no longer significant (*P*=0.10), but remained significant for SGA defined by the 5^th^ centile (*P*=0.004).

Rates of major congenital malformations and adverse outcomes did not differ significantly between the groups. Infants of women with unplanned pregnancies were significantly more likely to be admitted to a neonatal intensive care unit (*P*<0.001), a finding which remained significant after adjustment for covariates. A significantly higher proportion of infants from unplanned compared with planned pregnancies remained in hospital for > 10 days; *P=*0.006). This difference also remained significant after adjustment for covariates.

## Discussion

The majority of research to date has focused on the association of pre‐pregnancy care with pregnancy outcomes in women with diabetes, with only a small number of studies investigating pregnancy planning 18, 19. The present study showed that almost 40% of women with Type 1 diabetes did not plan their pregnancy, with only a quarter of women reporting having received preconception counselling. Younger women and those of lower social class were significantly less likely to plan their pregnancies. Unplanned pregnancy was associated with higher HbA_1c_ levels prior to and throughout pregnancy, lower folic acid uptake, more smoking in pregnancy and higher rates of SGA infants, infant neonatal intensive care unit admission and neonatal hospital stay exceeding 10 days. Previous studies in the general population have similarly found that women with an unplanned pregnancy were more likely to be younger, classed as socially deprived, and to engage in detrimental behaviours, including smoking 1, 2, 20, and were also more likely to have adverse outcomes 18.

With regard to HbA_1c_ levels in pregnancy, evidence has shown that higher levels are associated with a number of adverse outcomes, including congenital malformations, macrosomia and pre‐eclampsia 21, 22. In the present study, although women with unplanned pregnancies had significantly higher HbA_1c_ levels before and throughout their pregnancies, rates of outcomes such as those mentioned, were not significantly higher when compared to women with planned pregnancies. Smoking has previously been shown to reduce the risk of pre‐eclampsia 23 and also to increase the risk of SGA infants 24, and so may explain similar rates of pre‐eclampsia and LGA infants in the present study. The adjustment for smoking status in our logistic regressions, however, did not suggest that it had much of a confounding effect.

The proportion of women who reported receiving pre‐pregnancy counselling was significantly lower among those with unplanned pregnancies. It is important that all women with diabetes receive advice about pregnancy planning, with previous evidence showing a reduction in adverse outcomes associated with pre‐pregnancy care 11; however, it is interesting to note the similar rates of alcohol consumption between the two groups, and that a small proportion of women with unplanned pregnancies were taking folic acid prior to pregnancy, which may demonstrate that some messages are getting through. Those who attend pre‐pregnancy counselling/care are a self‐selecting group, so other more pragmatic solutions may be needed. These may include education about relationships and safe sex, and access to safe, effective contraception, particularly for women of lower social class.

It is unclear why women in the present study did not plan their pregnancies, but previous research suggests that both women and healthcare professionals are not aware of the full range of risks and complications associated with diabetes in pregnancy 10, 25, 26. It is important that pregnancy planning is seen as a shared responsibility among women, their partners, their families and their healthcare providers. Further research is needed to determine the extent to which these groups are aware of the risks and complications associated with diabetes in pregnancy.

The present study has a number of strengths. Firstly, DAPIT includes one of the largest contemporary datasets of women with Type 1 diabetes, with 762 women from across the UK. The study comprises a well characterized cohort of women with Type 1 diabetes, with extensive information being collected about pregnancy and pregnancy outcomes. Secondly, the study protocol included details of an extensive number of maternal and neonatal outcomes in these women.

The study also has a number of limitations. Participants in the study were those who consented to take part and, as a result, they may not be truly representative of the total population of women with diabetes. Pregnancy planning was a self‐reported response and not all participants may have answered honestly, which could have led to a bias towards under‐reporting of unplanned pregnancies. Additionally, it is possible that women biased their answers in relation to other questions, such as questions surrounding the use of alcohol in pregnancy. The study was also unable to determine if women specifically received specialized pre‐pregnancy care or pre‐pregnancy counselling as it was not possible to relate the data back to pre‐pregnancy care clinics. It is therefore likely that those who reported having received preconception counselling represent a mixture of women who received counselling and specialized pre‐pregnancy care.

In conclusion, although pregnancy planning did not affect the rates of all maternal and neonatal outcomes, unplanned pregnancy was associated with higher rates of SGA infants, admission to a neonatal intensive care unit and a longer neonatal stay in hospital. This study showed an association between lower social class and unplanned pregnancy in women with Type 1 diabetes. It is important that the risks associated with diabetes in pregnancy are highlighted to all women, their partners and families, and also to healthcare professionals. In addition to this, women, particularly those from lower social classes, should have access to good sex education and safe effective contraception, which may go some way to reducing unplanned pregnancies. Further research is required to determine the awareness among both women with diabetes and their healthcare professionals of the risks associated with diabetes in pregnancy.

## Funding sources

DAPIT was funded by grants 064028/Z/02/Z and 083145/Z/07/Z from the Wellcome Trust (registered charity no. 210183). A.C.W.'s PhD studentship was funded by the Department of Employment and Learning, Northern Ireland.

## Competing interests

None declared.
